# Nematode-Derived Proteins Suppress Proliferation and Cytokine Production of Antigen-Specific T Cells via Induction of Cell Death

**DOI:** 10.1371/journal.pone.0068380

**Published:** 2013-06-21

**Authors:** Wiebke Hartmann, Yannick Brenz, Manchang Tanyi Kingsley, Irene Ajonina-Ekoti, Norbert W. Brattig, Eva Liebau, Minka Breloer

**Affiliations:** 1 Bernhard Nocht Institute for Tropical Medicine, Hamburg, Germany; 2 Institute of Agricultural Research for Development, Veterinary Research Laboratory Wakwa Regional Center, Ngaoundere, Cameroon; 3 Institute of Animal Physiology, University of Münster, Münster, Germany; Wayne State University, United States of America

## Abstract

In order to establish long-lasting infections in their mammalian host, filarial nematodes have developed sophisticated strategies to dampen their host’s immune response. Proteins that are actively secreted by the parasites have been shown to induce the expansion of regulatory T cells and to directly interfere with effector T cell function. Here, we analyze the suppressive capacity of *Onchocerca* *volvulus* -derived excreted/secreted proteins. Addition of two recombinant *O* *. volvulus* proteins, abundant larval transcript-2 (*Ov*ALT-2) and novel larval transcript-1 (*Ov*NLT-1) to cell cultures of T cell receptor transgenic CD4^+^ and CD8^+^ T cells suppressed antigen-specific stimulation *in vitro*. Ovalbumin-specific CD4^+^ DO11.10 and OT-II T cells that had been stimulated with their cognate antigen in the presence of *Ov*ALT-2 or *Ov*NLT-1 displayed reduced DNA synthesis quantified by ^3^H-thymidine incorporation and reduced cell division quantified by CFSE dilution. Furthermore, the IL-2 and IFN-γ response of ovalbumin-specific CD8^+^ OT-I T cells was suppressed by *Ov*ALT-2 and *Ov*NLT-1. In contrast, another recombinant *O* *. volvulus* protein, microfilariae surface-associated antigen (*Ov*103), did not modulate T cell activation, thus serving as internal control for non-ESP-mediated artifacts. Suppressive capacity of the identified ESP was associated with induction of apoptosis in T cells demonstrated by increased exposure of phosphatidylserine on the plasma membrane. Of note, the digestion of recombinant proteins with proteinase K did not abolish the suppression of antigen-specific proliferation although the suppressive capacity of the identified excreted/secreted products was not mediated by low molecular weight contaminants in the undigested preparations. In summary, we identified two suppressive excreted/secreted products from *O* *. volvulus* , which interfere with the function of antigen-specific T cells *in vitro*.

## Introduction

It is estimated that worldwide more than 1.5 billion people are at risk of being infected with filarial nematodes. *Wuchereria* *bancrofti* , *Brugia malayi* and *Onchocerca* *volvulus* are the causative agents of chronic diseases such as lymphatic filariasis and river blindness [1] and cause a major public health problem. These long-lived parasites have evolved sophisticated strategies to evade the immune response of their host and to survive in the host for more than a decade [2].

The life cycle of filarial worms in humans and in different animal models is comparable, starting in an arthropod as intermediate host. First stage larvae so called microfilariae (MF) are taken up during a blood meal from a mosquito, blackfly or blood-sucking mite. Within the vector the MF undergo two molts und develop into infective third stage larvae (L3). L3 are transmitted during a second blood meal to their final host. Here, L3 migrate depending on the species to different sides of the body. *O* *. volvulus* adults reside in nodules in the subcutaneous tissue while *W* *. bancrofti* and *B. malayi* adults dwell in lymphatic vessels. Different developmental stages have adapted to different niches in the body but utilize similar strategies to promote their survival in their living environment. Chronic helminth infections induce a regulatory network, which is composed of regulatory T cells, alternatively activated macrophages, and anti-inflammatory cytokines [3]. These suppressive cell types might compromise the immune response to the parasite and to unrelated antigens such as vaccines [4]. Impaired proliferation of peripheral T cells to filarial-specific antigens, a phenomenon called lymphocyte hypoproliferation, was already shown in filarial infected humans [5]. Beyond that, pre-existing filarial infection interferes with cellular and humoral immune response to vaccinations such as tetanus toxoid vaccination [6–8].

Using the murine model of human filarial infection, *Litomosoidessigmodontis* , we have shown recently that concurrent nematode infection suppressed the response to model antigen immunizations and to an experimental vaccination against *Plasmodium berghei* infection in mice [9,10]. Thereby, *L* *. sigmodontis* infection interfered with both, vaccine-induced activation of CD4^+^ T helper cells and cytotoxic CD8^+^ T cells *in vivo*. In general, this helminth-induced immunosuppression depends most likely on the living parasite as drug treatment restored the T cell response [11]. Interaction of helminths with the immune system of their host seems to be mediated by soluble molecules, namely excretory/secretory products (ESP), which are released by live parasites. ESP are biologically active proteins that are either actively exported through secretory pathways or simply leak from the parasite surface [12]. To dissect the composition of ESP, supernatants were collected from *in vitro* cultured worms and subsequently analyzed by mass spectrometry. Interestingly, a great similarity concerning the protein sequences have been shown for different filarial species such as *B. malayi* and *O* *. volvulus* [13]. Larval stage-specific expression of different proteins was shown for *O* *. volvulus* without analyzing the function of these proteins in detail [14,15].

The aim of our study was to analyze selected proteins secreted by *O* *. volvulus* in order to identify proteins with immunomodulatory capacity. In this context, ESP derived from the parasitic nematode *Heligmosomoidespolygyrus* have been shown to induce Foxp3^+^ regulatory T cells *in vitro* and *in vivo* [16] and recombinant cystatin derived from *Acantocheilonema* vitae suppressed effector T cell function *in vitro* [17] and *in vivo* [18,19]. Recombinant *O* *. volvulus* proteins were generated and tested in an *in vitro* proliferation assay employing T cell receptor (TCR) transgenic T cells. We identified two ESP from *O* *. volvulus* that suppressed proliferation of ovalbumin-specific T cells. *Ov*ALT-2 and *Ov*NLT-1 diminished proliferation and cytokine secretion of model antigen-specific T cells *in vitro* while another recombinant ESP, *Ov*103, displayed no suppressive capacity. We ruled out contaminating endotoxin or toxic low molecular contaminants as suppressive elements in the recombinant ESP preparations. Furthermore, suppressed proliferation was associated with increased induction of cell death but was resistant to proteinase K treatment. Taken together, we identified two new *O* *. volvulus* -derived proteins that suppress the function of model antigen-specific T cells *in vitro.*

## Materials and Methods

### *Mice*

Animal experimentation was conducted at the animal facility of the Bernhard Nocht Institute for Tropical Medicine in agreement with the German animal protection law under the supervision of a veterinarian. The experimental protocols have been reviewed and accepted by the responsible federal health Authorities of the State of Hamburg, Germany, the "Behörde für Gesundheit und Verbraucherschutz" permission number 98/11. Mice were sacrificed by cervical dislocation under CO_2_ narcosis. BALB/c mice were purchased from Charles River. OT-II, OT-I and DO11.10 mice were bred in the animal facility of the Bernhard Nocht Institute. Female and male mice were 8-12 weeks of age.

### *Reagents and antibodies*

Anti-CD4-allophycocyanin (clone RM4-5) and Annexin V PE Apoptosis Detection Kit were purchased from eBioscience; CFSE was obtained from Invitrogen (Carlsbad, CA, USA). OVA_323-339_ was obtained from JPT Peptide Technologies GmbH (Berlin, Germany) and OVA_257-264_ was purchased from MWG Biotech (Ebersberg, Germany).

### *Preparation of cDNA from adult O. volvulus*

Onchocercomas were derived from studies conducted in Liberia and Ghana [20,21]. Whole worm RNA was prepared from *O* *. volvulus* using TRIzol reagent (Invitrogen, Carlsbad, CA, USA) according to the supplier’s protocol. First strand cDNA synthesis was then performed using 2 µg of RNA as template and oligo (dT)_18_ primers, following the manufacturer’s protocol (Thermo Scientific). Coding sequences were then amplified by PCR using Phusion^®^ High-Fidelity DNA Polymerase (New England Biolabs) following the manufacturer’s instructions.

### *Cloning, expression and purification of O. volvulus candidate antigens OvNLT-1, OvALT-2, Ov103, and Ov7*

Primers used for PCR are listed in Table S1. Following amplification, the PCR products and the expression vector pJC40 [22] were digested using appropriate FastDigest® restriction enzymes (Thermo Scientific) and ligated using T4 DNA ligase (Invitrogen). 5 µl of each ligation were transformed into XL10-gold ultra competent cells according to the supplier’s protocol (Stratagene). Positive clones were identified by test digestion and sequencing. After transformation of the respective expression plasmids into *E. coli* Rossetta gami DE3 cells (*Ov*NLT-1) or *E. coli* BL21DE3 Star cells (*Ov*ALT-2, *Ov*103, *Ov*7) (Stratagene), expression of the tagged proteins was initiated by the addition of iso-propyl-beta-D-thiogalactopyranoside (IPTG) (0.05 mM IPTG for *Ov*NLT-1, 0.5 mM IPTG for *Ov*ALT-2 and 1 mM IPTG for *Ov*7) once the cultures had reached A_600_ = 0.5 (*Ov*ALT-2, *Ov*7) or A_600_ = 0.2(OvNLT-1). Cells were left to grow for additional 3 h (*Ov*7) or overnight (*Ov*NLT-1, *Ov*ALT-2) at 37^°^C. Expression of *Ov*103 was carried out by autoinduction [23]. Cells were harvested by centrifugation and the resulting bacterial pellets were resuspended in lysis buffer (50 mM Tris, 500 mM NaCl, 10% glycerol, 0.1% (v/v) Triton X-100, 10 mM imidazole, 1 mM phenylmethylsulfonyl fluoride pH 8.0) for purification of *Ov*103 and *Ov*7. For purification of *Ov*NLT-1 and *Ov*ALT-2 dithiothreitol (5 mM) was added to the lysis buffer. Pellets were sonicated using a digital sonifier set to 30 watts and 30% amplitude (Branson). The resulting lysate was centrifuged at 10,000 x g for 30 minutes and the supernatant was purified by affinity column chromatography with profinity^TM^ IMAC Ni^2+^-nitrilotriacetic acid resin (Bio-Rad Laboratories, Germany). Columns were washed with 25 column volumes of washing buffer (lysis buffer containing 20 mM imidazole and 5 mM DTT). The recombinant proteins were eluted with lysis buffer containing 300 mM imidazole (*Ov*NLT-1, *Ov*ALT-2, *Ov*7) or 250 mM imidazole (*Ov*103) and dialysed in 20 mM Tris buffer, pH 7.5, containing 150 mM NaCl, 0.2 mM DTT, prepared with LPS free water (Aqua B. Braun, Melsungen AG, Germany). The purity of the dialysed proteins was controlled by resolution on 12.5% SDS PAGE and Coomassie blue staining. Proteins were concentrated using Millipore 10,000 MWCO (Amicon Ultra) (6000–8000 MWCO for *Ov*ALT-2) and the concentration was determined by the method of Bradford [24]. 60 µg/mL of polymyxin B was added to all stages of purification and the purified proteins and contamination with LPS was quantified using LAL Chromogenic Kit ( *Limulus* Amebocyte Lysate; QCL-1000, Lonza, Walkersville, MD, USA). For proteinase K digestion, 0.05 µg proteinase K per µg recombinant protein were added at the beginning and again after 30 min incubation at 56°C. The digestion was stopped after 60 min by incubation at 75°C for 20 min and 95°C for 20 min. To generate a 3 kDa filtrate the recombinant proteins were centrifuged at 4000 x g using Amicon Ultra-4 Centrifugal Filter Units (3 kDa). The resulting filtrate was checked for remaining protein via SDS-PAGE and Coomassie blue-staining.

### *In vitro stimulation of lymphocytes and analysis of OVA-specific T cell proliferation*

Splenocytes (2 x 10^5^ / well) were cultivated in 96 well round bottom plates for 72 h at 37°C and 5% CO_2_ in RPMI 1640 medium supplemented with 10% fetal calf serum, L-glutamine (2 mg/mL), HEPES (20 mM) and gentamycin (50 µg/mL). For stimulation, cells were either incubated with medium alone or with to 10 ng/mL OVA_323-339_ or OVA_257-264_ peptide alone or in the presence of ESP (2.5–10 µg/mL), digested ESP or 3 kDa filtrate in triplicates. Apoptosis was measured 6 h after *in vitro* stimulation of spleen cells in the presence of ESP and the cognate antigen. Concentration of IL-2 and IFN-γ in the supernatant of *in vitro* stimulated OT-I spleen cells was quantified after 72 h culture employing DuoSet ELISA development system kits (R&D Systems, Wiesbaden, Germany) according to the manufacturer´s instructions. Proliferation after 72 h culture of DO11.10 or OT-II spleen cells was either measured by uptake of ^3^H-thymidine for additional 18 h culture or as carboxyfluorescein diacetate succinimidylester (CFSE) dilution as described before [9]. As CFSE dilution is not as sensitive as ^3^H-thymidine incorporation we increased the concentration of OVA_323-339_ peptide 5-fold in order to measure CFSE dilution *in vitro* (data not shown). For CFSE labeling 5 x 10^7^ spleen cells were resuspended in 10 mL sterile PBS. After addition of 200 µL 50 µM (for *in vitro* proliferation) or 500 µM (for *in vivo* proliferation) CFSE cells were incubated for 10 min at 37°C. Labeling reaction was stopped by addition of 40 mL 3% FCS in PBS and cells were washed thrice. For *in vitro* proliferation CFSE-labeled cells were incubated as described in the previous section. For *in vivo* proliferation CFSE labeled transgenic OT-II spleen cells (1 x 10^7^) were injected intravenously into C57BL/6 mice as recipients. The day following transfer mice received 50 µg OVA_332-339_ i.p. in PBS. Half of the mice received an additional injection of 20 µg *Ov*7 i.p. at the day of the adoptive transfer and one day later. Mice were sacrificed 48 h later, spleen cells isolated and stained with anti-CD4 antibody. Number of proliferation cycles was calculated by CFSE dilution.

### *Flow cytometry*

Surface staining of spleen cells was performed using anti-CD4 allophycocyanin antibody as described previously [25]. Cell death was analyzed by 7-AAD incorporation and Annexin V phycoerythrin staining according to the manufacturer´s instructions. Samples were analyzed on a FACS Calibur flow cytometer (Becton Dickinson, Mountain View, CA, USA).

### *Statistical analysis*

Statistical analysis was performed with GraphPad Prism Software (San Diego, USA) using 2-way ANOVA with Bonferroni post-test or students t test. A p-value of < 0.05 was considered to be statistically significant.

## Results

### *Suppression of antigen-specific T cell activation by O. volvulus proteins*

**Figure 1:**
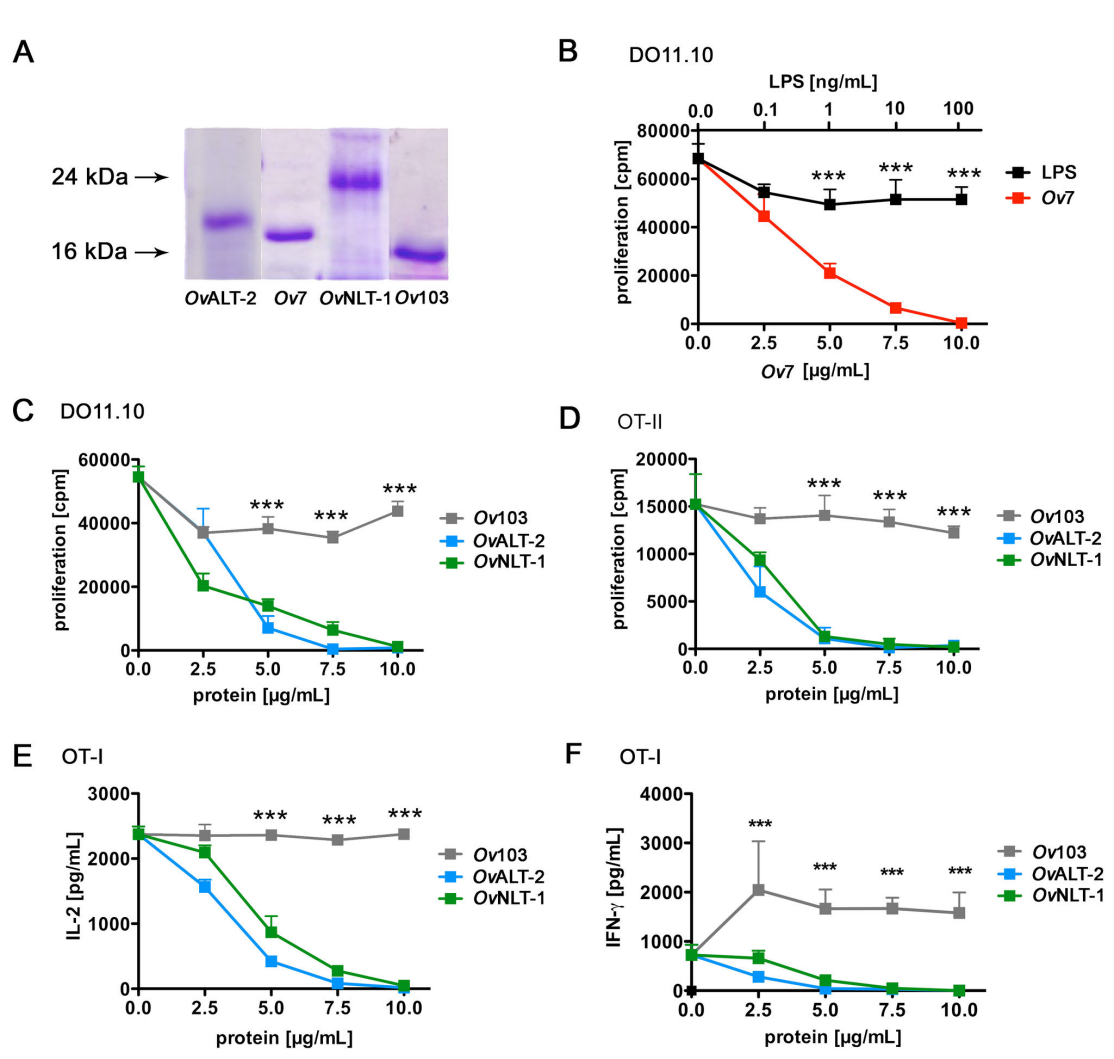
Nematode-derived ESP suppress OVA-specific T cell function. A) Coomassie blue-staining of recombinant proteins after purification and separation by SDS-PAGE. B) Spleen cells from TCR transgenic DO11.10 (B, C), OT-II (D) or OT-I (E, F) mice were stimulated with 10 ng/mL OVA_323-339_ (B-D) or OVA_257-264_ (E, F) peptide in the presence of ESP or LPS in increasing concentrations as indicated on the x-axis. OVA-specific proliferation of DO11.10 (B, C) and OT-II (D) T cells was measured after 72 h as ^3^H-thymidine incorporation. IL-2 (E) and IFN-γ (F) secretion by OT-I T cells was analyzed by ELISA. Error bars show SD of triplicates. Data shown are representative for five (B, C) or two (D, E, F) independent experiments. Asterisks indicate significant differences in the mean of *Ov*103 or LPS to *Ov*7 or *Ov*ALT-2 or *Ov*NLT-1 (*** p < 0.001).

To analyze the immunomodulatory capacity of *O* *. volvulus* -derived ESP we generated a set of different recombinant proteins in *E. coli* and purified the proteins via their N-terminal histidine tag. In order to control purity of the protein suspensions SDS-PAGE was performed (Figure 1A).

Suppressive capacity of the recombinant *O* *. volvulus* -derived ESP was analyzed in an *in vitro* proliferation assay using TCR transgenic mice. In DO11.10 mice all T cells are specific for the OVA_323-339_ peptide associated with major histocompatibilty complex II (MHC-II) I-A^d^ [26]. To verify an impact of *Ov*ESP on antigen-specific T cell activation DO11.10 spleen cells were stimulated with OVA_323-339_ in the presence of increasing concentrations of ESP. Proliferation was quantified as DNA synthesis, i.e. ^3^H-incorporation and was suppressed by co-incubation with the previously described *Ov*7 [17] (Figure 1B). In addition *Ov*ALT-2 and *Ov*NLT-1 suppressed proliferation while *Ov*103 did not modulate OVA-specific proliferation of DO11.10 T cells (Figure 1C). To demonstrate suppression of T cells carrying a different transgenic TCR, we analyzed antigen-specific proliferation of splenocytes derived from OT-II mice. These mice were generated on the C57BL/6 background and are transgenic for a MHC class II (I-A^b^) restricted OVA_323–339_-specific TCR [26]. Regardless of the genetic background of the T cells, *Ov*ALT-2 and *Ov*NLT-1 but not *Ov*103 suppressed DNA synthesis of OVA-stimulated spleen cells in a dose-dependent manner (Figure 1D).

As *Ov* proteins were expressed in *E. coli*, ESP preparations might be contaminated with low amounts of bacterial pathogen-associated molecular patterns (PAMP) such as lipopolysaccharide (LPS). Although suppressive and non-suppressive proteins were prepared in parallel we formally wanted to rule out side effects due to endotoxin contamination. Endotoxin activity as measured by *Limulus* amebocyte lysate assay was maximally 2.4 EU per µg protein and was comparable in suppressive and non-suppressive protein preparations. We added LPS in increasing concentrations to spleen cell cultures derived from DO11.10 mice and measured OVA-specific T cell proliferation. LPS in the range of 0.1 ng/mL to 100 ng/mL corresponding to 1 EU/mL to 1000 EU/mL did not suppress proliferation of T cells while increasing concentrations of *Ov*7 abolished T cell proliferation *in vitro* (Figure 1B).

In addition to the interference of nematode-derived proteins with proliferation of TCR transgenic CD4^+^ T cells we analyzed the impact on cytokine production by cytotoxic CD8^+^ T cells. For this purpose, T cells from TCR transgenic OT-I mice that are specific for OVA_257-264_-peptide in association with MHC class I (H-2K^b^) were used [27]. Antigen-specific activation of OT-I spleen cells was measured as IL-2 and IFN-γ cytokine secretion. Addition of both, *Ov*ALT-2 and *Ov*NLT-1, decreased secretion of IL-2 and IFN-γ compared to addition of *Ov*103 as negative control protein (Figure 1E,F). Taken together, we identified *Ov*ALT-2 and *Ov*NLT-1 as ESP that suppress proliferation and cytokine secretion by OVA-specific CD4^+^ T cells and CD8^+^ T cells derived from TCR transgenic mice.

**Figure 2:**
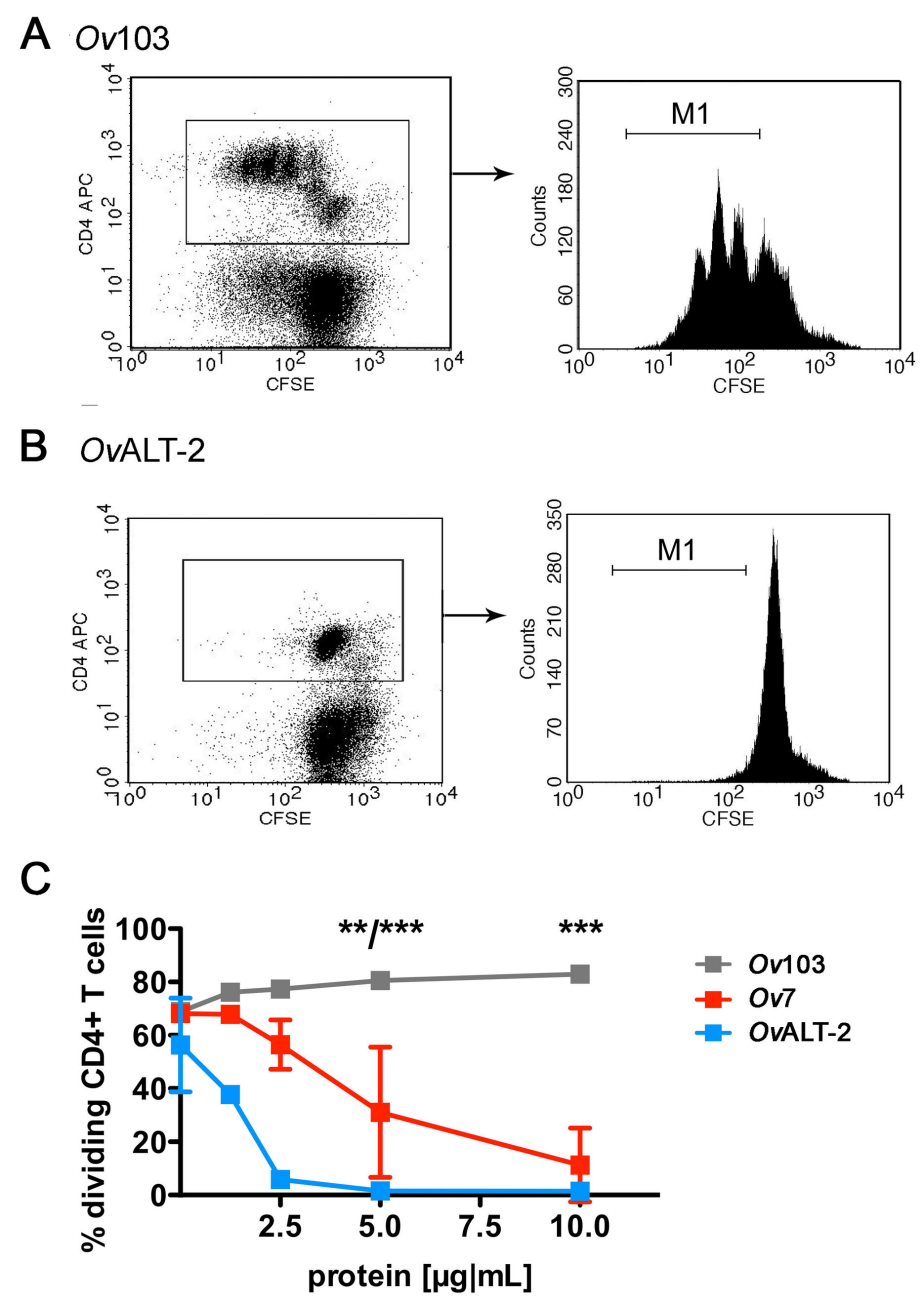
*Ov*ALT-2 suppresses OVA-specific OT-II T cell proliferation *in* *vitro.* Spleen cells were isolated from OT-II mice and labeled with CFSE. Cells were stimulated with 50 ng/mL OVA_323-339_ peptide in the presence of nematode-derived proteins *Ov*103, *Ov*ALT-2 or *Ov*7 in increasing concentrations as indicated on the x-axis. After 72 h cells were stained with anti-CD4 mAb and proliferation of CD4^+^CFSE^+^ T cells was analyzed by flow cytometry as percentage of dividing T cells (marker M1). Shown is a representative dot blot of OT-II spleen cells after incubation with 7.5 µg/mL *Ov*103 (A) or *Ov*ALT-2 (B) in the presence of OVA-peptide. C) Graph shows percentage of dividing T cells in the presence of indicated concentration of ESP. Data are presented as the mean of combined results derived from two independent experiments, error bars show SEM. Asterisks indicate significant differences in the mean of *Ov*103 to *Ov*7 or *Ov*ALT-2, respectively (** p < 0.01; *** p < 0.001).

To directly measure proliferation in addition to DNA synthesis, we labeled OT-II cells with the fluorescent dye CFSE that is diluted upon cell division. After stimulation in the presence of the tested ESP, we gated on CD4^+^ OT-II T cells and visualized dividing cells by their decreasing CFSE content (Figure 2AB). Thereby we calculated the proportion of dividing cells upon antigen-specific stimulation in the presence of *Ov*7, *Ov*ALT-2 and *Ov*103 as negative control protein (Figure 2C). *Ov*ALT-2 and *Ov*7 suppressed the antigen-driven division of OVA-specific OT-II T cells whereas *Ov*103 again did not affect T cell function.

### *Limited impact of Ov7 on the clonal expansion of TCR transgenic T cells in vivo*

**Figure 3:**
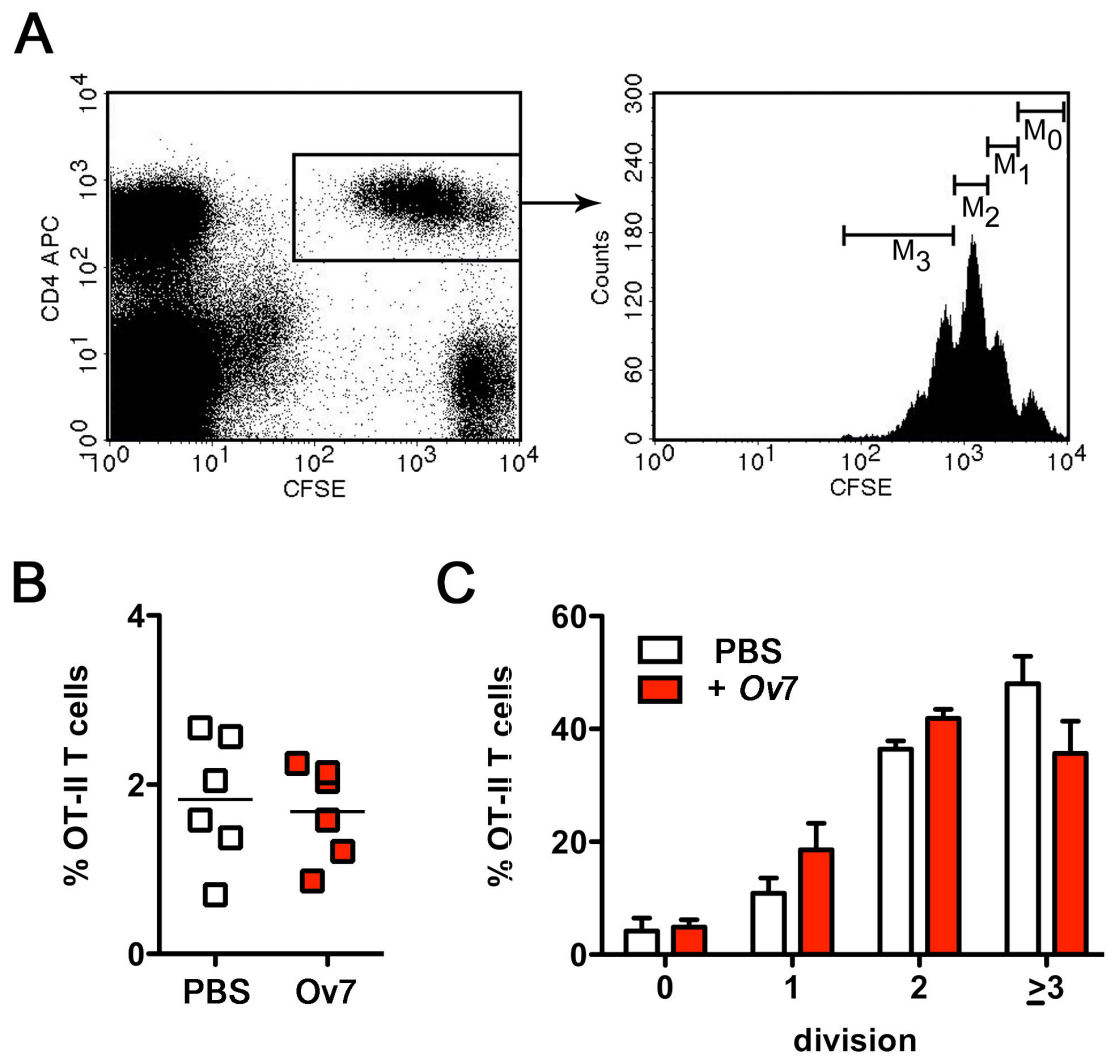
No impact of *Ov*7 on proliferation of OT-II T cells *in vivo*. CFSE-labeled OT-II spleen cells were adoptively transferred i.v. into C57BL/6 mice. 20 µg *Ov*7 was injected i.p. at the day of the transfer and one day later. Mice were sacrificed 2 d after *in vivo* activation with 50 µg OVA_323-339_ peptide and proliferation was analyzed. A) Representative dot blot showing the gating strategy. We gated on CD4^+^CFSE^+^ cells to calculate the frequency of OT-II T cells in the spleen (B) and analyzed the number of cell divisions OT-II T cells have undergone (C). Shown is the frequency of OT-II T cells that did not divide (M_0_) or divided once (M_1_), twice (M_2_), or three times and more (M_3_) after stimulation in the presence of PBS (white bars) or *Ov*7 (red bars). Shown are combined results from two independent experiments with six mice per group, error bars show SEM. Analysis with students t test revealed no significant statistical difference of the mean (p = 0,73 (B) and p = 0,13 (C, three and more division cycles). This result was reproduced in two other independent experiment using five control and seven *Ov*7-treated mice.

We have shown before that concurrent infection of mice with the filarial nematode *L* *. sigmodontis* reduced proliferation of OVA-specific OT-II T cells *in vivo* [9]. Having identified filarae-derived ESP that suppressed OVA-specific T cell proliferation *in vitro*, we next wanted to address if these ESP would also suppress T cell proliferation *in vivo* i.e. mimic the suppressive effect of the ongoing filarial infection. Therefore we adoptively transferred OT-II T cells to syngenic C57BL/6 mice that were treated with 20 µg *O*v7 or PBS at the day of the adoptive transfer and one day later. OT-II T cells were stimulated *in vivo* by injection of the cognate antigen OVA_323-339_ one day after adoptive transfer and frequencies of OT-II T cells in the spleen were calculated two days later (Figure 3A). *Ov*7 treatment did not reduce the overall proportion of OT-II T cells in the spleen compared to untreated mice (Figure 3B, p = 0,73). *Ov*7 treatment slightly interfered with the number of cell divisions undergone by OT-II T cells *in vivo* (Figure 3C). By trend, a reduced number of OT-II T cells divided three times or more in mice that had been treated with *Ov*7 compared to untreated mice (p= 0,13). As we did not record statistically significant changes in *in vivo* T cell proliferation by treatment with the well-defined immunosuppressive ESP *Ov*7 that we employed as a positive control, we did not repeat these experiments with the undefined new ESP.

### *OvALT-2- and OvNLT-1-mediated inhibition of OVA-specific proliferation is resistant to proteinase K digestion*

**Figure 4:**
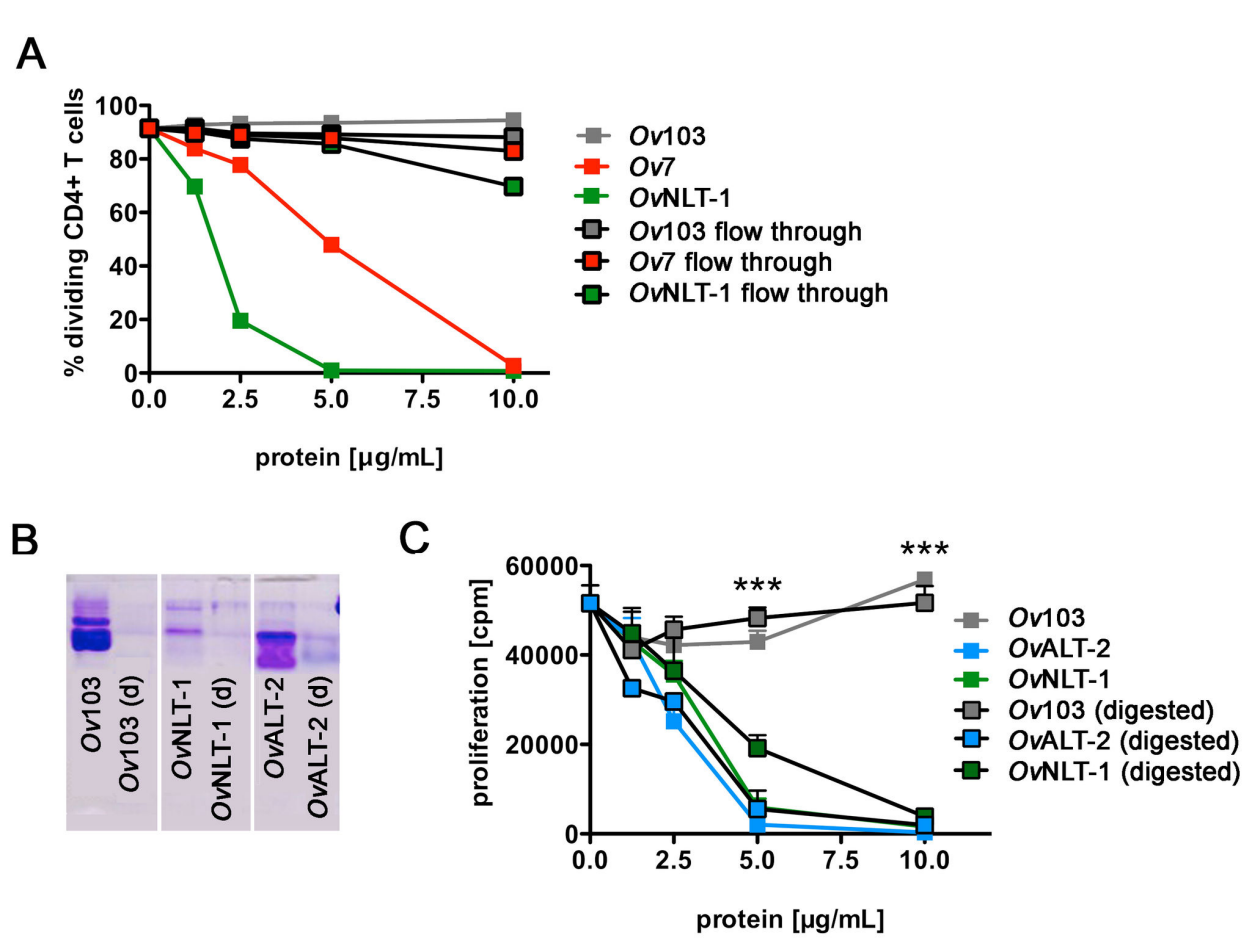
Suppression of OVA-specific T cell proliferation by proteinase K digested *O*. *vovlvulus* ESPs. A) ESPs were separated using a 3 kDa Amicon filter into the protein and the "flow through" containing molecules smaller than 3 kDa. Spleen cells derived from DO11.10 mice were labeled with CFSE and stimulated with 10 ng/mL OVA_323-339_ peptide in the presence of ESP or "flow through". Proliferation was analyzed after 72 h by flow cytometry and is depicted as frequency of dividing CD4^+^ T cells. B) SDS-PAGE and Coomassie blue staining of recombinant nematode-derived proteins that were either native or digested with proteinase K. C) Thymidine incorporation of DO11.10 cells after incubation with native or proteinase K digested nematode-derived proteins. Shown are results from one experiment representative for two independent repeats. Error bars show SEM of triplicates and asterisks indicate significant differences of the mean (*** p < 0.001).

Working with proteins that have been expressed in bacteria heat-inactivation is a common control to dissect endotoxin-mediated from protein-mediated effects. To our surprise, heat-inactivation did not abolish the suppressive capacity of the investigated ESP (data not shown and Figure 4C). This may either suggest that suppression was not caused by the ESP but by other constituents in the recombinant protein preparation, or suppressive ESP may function in a heat-stable manner. In order to rule out that decreased proliferation was mediated by low molecular weight *E. coli*-derived contaminants we separated ESP using a 3 kDa Amicon filter. *Ov*7 and *Ov*NLT-1 protein preparations (>3 kDa) reproducibly suppressed proliferation of OVA-specific T cells while *Ov*103 and the low molecular filtrate of all proteins did not alter proliferation (Figure 4A). Thus, we could exclude that toxic low molecular bacterial contaminants in the preparation caused the observed interference with antigen-specific proliferation.

To test if the suppression of OVA-specific proliferation by nematode-derived proteins *Ov*ALT-2 and *Ov*NLT-1 was dependent on the intact protein, we treated proteins with proteinase K. Proteinase K activity in the samples was terminated by heat-inactivation after digestion. Complete digestion of the proteins was confirmed by Coomassie-staining after SDS PAGE (Figure 4B). Unexpectedly, the digested preparations of *Ov*ALT-2 and *Ov*NLT-1 still suppressed the OVA-specific proliferation of DO11.10 T cells to the same extent as the corresponding amount of undigested protein (Figure 4C). This T cell suppression by degraded *Ov*ALT-2 and *Ov*NLT-1 proteins was not due to cytotoxic products or residual proteinase activity in the reaction as proteinase K treated *Ov*103 did not suppress DO11.10 T cell proliferation. Therefore, suppression was mediated by heat-stable small fragments of *Ov*NLT-1 and *Ov*ALT that were not degraded by proteinase K digestion.

### *OvALT-2 and OvNLT-1 induce apoptosis in spleen cells*

**Figure 5:**
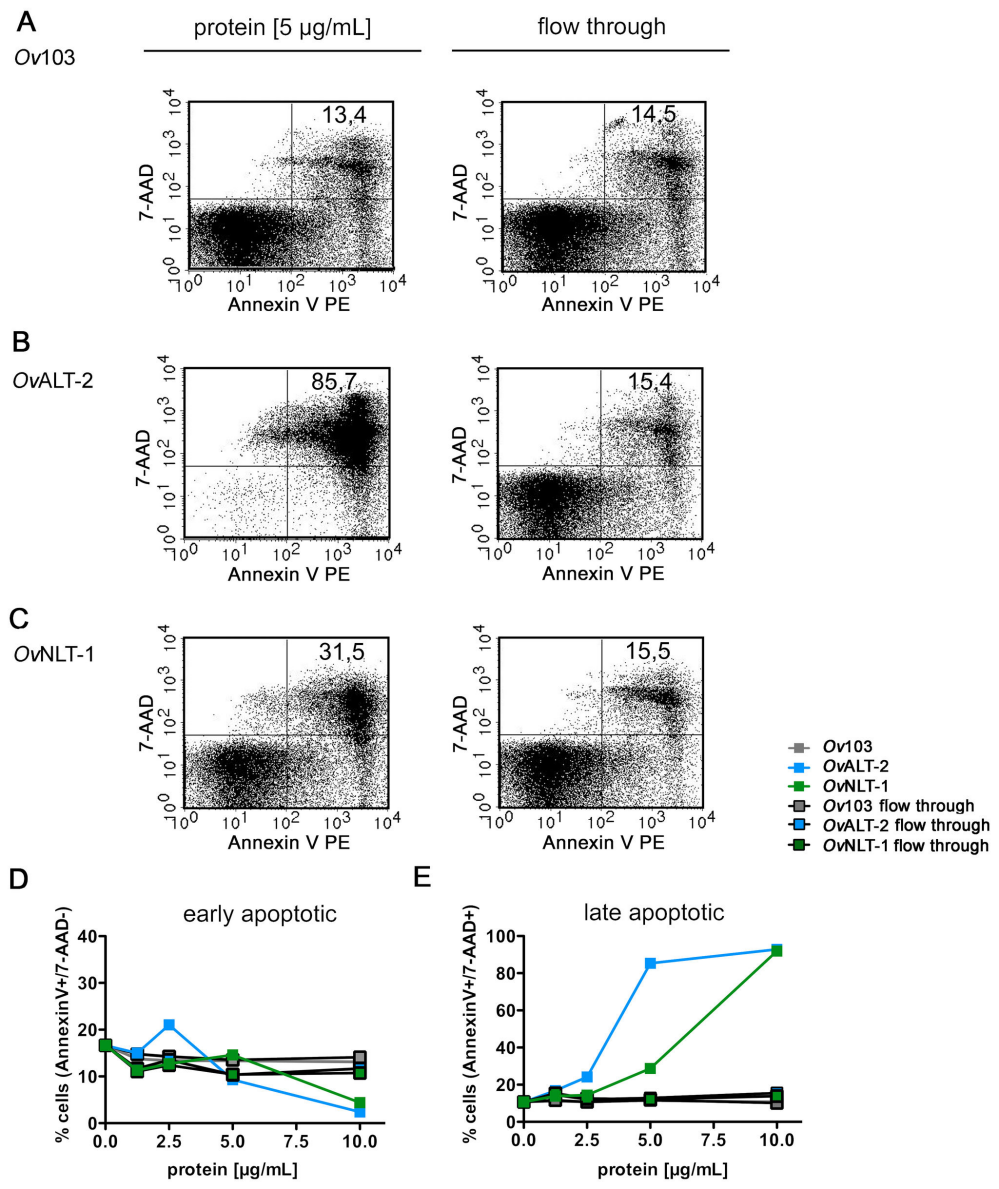
Nematode-derived proteins *Ov*NLT-1 and *Ov*ALT-2 induce apoptosis in spleen cells. ESP were separated into protein and "flow through" using Amicon filter. Spleen cells were isolated from BALB/c mice and incubated with increasing concentration of *Ov*103, *Ov*ALT-2 and *Ov*NLT-1 or the corresponding low molecular weight filtrate ("flow through"). After 6 h of incubation cells were stained with 7-AAD and annexin V. Apoptotic cells (annexin V^+^) were further identified by 7-AAD expression as early apoptotic cells (7-AAD^-^) or late apoptotic (7-AAD^+^). Representative dot blots are shown for spleen cells after incubation with 5 µg/mL of nematode-derived *Ov*103 (A) as control peptide, *Ov*ALT-2 (B) or *Ov*NLT-1 (C). Statistical analysis of the frequencies of early (D) and late (E) apoptotic cells. Results show one individual experiment and are representative for two independent experiments.

In order to analyze the underlying mechanisms of suppression we measured induction of apoptosis by nematode-derived proteins. To distinguish necrosis and apoptosis, spleen cells were stained with 7-aminoactinomycin (7-AAD) and annexin V. One early feature of apoptosis is indicated by the loss of plasma membrane symmetry and exposure of phosphatidylserine at the outer membrane. Annexin V is a phospholipid-binding protein, which binds to phosphatidylserine on the plasma membrane of early apoptotic cells. Ongoing apoptosis is characterized by complete loss of membrane integrity, allowing intercalation of vital dyes such as 7-AAD in the DNA. By means of 7-AAD and annexin V we distinguished between early apoptotic cells (annexin V^+^/7-AAD^-^) and late apoptotic or necrotic cells (annexin V^+^/7-AAD^+^). The frequency of apoptotic cells was determined after incubation with recombinant proteins or a 3 kDa filtrate of the same protein preparations to rule out effects induced by putative low molecular weight contaminants (Figure 5A–C). Neither 3 kDa filtrate (flow through) nor the proteins itself altered the proportion of early apoptotic cells in splenic cultures (Figure 5D). In contrast, cells incubated with 5-10 µg/mL *Ov*ALT-2 and *Ov*NLT-1 showed a higher proportion of late apoptotic cells after 6 h of incubation (Figure 5E). Thereby 10 µg/mL of *Ov*ALT-2 and *Ov*NLT-1 induced apoptosis in almost 100% of the spleen cell culture suggesting that the pro-apoptotic activity of ESPs was not cell type specific. Late apoptosis was not detectable in spleen cells cultured in the presence of the 3 kDa filtrate ruling out that toxic low molecular contaminants in *Ov*ALT-2 and *Ov*NLT-1 protein preparations induced apoptosis (Figure 5E). *Ov*103 did not induce cell death thus showing specificity of *Ov*ALT-2- and *Ov*NLT-1-induced suppression.

Taken together, our results identified *Ov*ALT-2 and *Ov*NLT-1 as nematode-derived proteins that mediate suppression of antigen-specific T cell activation. Suppressive capacity of the proteins was associated with induction of cell death but was resistant to proteinase K digestion.

## Discussion

In our previous studies we demonstrated that concurrent infection with the pathogenic nematodes *L* *. sigmodontis* and *Strongyloides* *ratti* suppressed the efficiency of vaccinations to bystander antigens *in vivo* [9,10,25]. Particularly infection of mice with *L* *. sigmodontis* resulted in drastically reduced antibody titer in response to vaccination with a thymus-dependent model antigen. Suppression of B cell response in nematode-infected mice was due to suppression of T helper cell expansion [9]. Moreover, we demonstrated reduced circumsporozoite-specific CD8^+^ cytotoxic T cell responses upon immunization with a *P. berghei*-specific vaccine in *L* *. sigmodontis* -infected mice compared to non-infected mice [10]. These results are in line with several studies describing the interference of ongoing nematode infections with T cell activation [28]. Screening for nematode products with the potential to suppress T cell activation, several candidates have been described [12,13]. Cystatins derived from *O* *. volvulus* and *Acanthocheilonema viteae* for instance suppressed proliferation of human peripheral blood lymphocytes *in vitro* [17] and ameliorated experimental asthma *in vivo* [18,19].

In the current study we explored the suppressive capacity of ESP derived from the human pathogen *O* *. volvulus* . In addition to the well-described *Ov*7 we identified two ESP that suppressed T cell function *in vitro*. *Ov*ALT-2 and *Ov*NLT-1 suppressed antigen-specific proliferation of two different MHC-II restricted TCR transgenic T cells, OT-II and DO11.10. Moreover, *Ov*ALT-2 and *Ov*NLT-1 suppressed antigen-specific IL-2 and IFN-γ production in spleen cell cultures derived from MHC-I-restricted OT-I mice thus indicating a downregulation of both CD4^+^ and CD8^+^ T cell function. Several *in vitro* studies using ESP from different helminth species suggest that one evasion strategy represents the induction of cell death in effector cells [29–32]. Recently, ESP from *Fasciola* *hepatica* have been shown to induce apoptosis in eosinophils, a cell type with a central role in the expulsion of helminths [29]. In line with these findings, we demonstrated the induction of cell death in spleen cell cultures upon *in vitro* exposure to *Ov*ALT-2 and *Ov*NLT-1 but not with *Ov*103.

Working with proteins that are expressed in *E. coli*, one major concern is a possible contamination with bacterial substances. PAMPs such as LPS might interfere with the activation of OVA-specific T cells. Addition of LPS containing the 10-100 fold endotoxic activity present in our ESP preparations did not modulate T cell proliferation indicating that suppressed proliferation was not due to LPS contamination. To further dissect artificial, *E. coli*-derived contaminant-mediated effects from protein-mediated suppression we included *Ov*103. This negative control protein was expressed similarly to the suppressive proteins *Ov*ALT-2 and *Ov*NLT-1 and did neither suppress proliferation nor induce cell death in T cells. To exclude suppression mediated by low molecular *E. coli*-derived toxic contaminants we used Amicon filtration to separate the protein from substances smaller than 3 kDa. Again control protein *Ov*103 and 3 kDa filtrate of the suppressive proteins *Ov*ALT-2 and *Ov*NLT-1 did not change proliferation or induce cell death.

Surprisingly, suppression of OVA-specific T cell proliferation was neither abolished by heat inactivation nor by digestion of the proteins with proteinase K. We controlled complete digestion of the proteins by SDS-PAGE thus ruling out that remaining intact ESP induced observed suppression of proliferation. We excluded interference of the digestion reaction itself with T cell activation, as the digested control protein did not modulate OT-II proliferation. Our results strongly suggest that small peptide fragments derived from *Ov*ALT-2 and *Ov*NLT-1 that remained intact after proteinase K digestion and that were not affected by heat-induced disruption of the tertiary structure of the protein would mediate suppression of T cell function. Along this lines suppression of allergic airway inflammation by application of ESP from *Heligmosomoidespolygyrus* during sensitization was not abolished by heat treatment [33]. Moreover, ESP derived from *Nippostrongylus* *brasiliensis* inhibited ovalbumin-induced allergy after heat inactivation and proteinase K digestion [34]. As these studies used an undefined ESP concentrate derived from *in vitro* nematode culture supernatants, the most likely explanation for heat-stable and proteinase-insensitive biologic activity was that immunosuppression was induced by lipids or carbohydrates instead of proteins. Our results, gained with purified recombinant proteins suggest small peptide fragments as additional mediator of proteinase-resistant bioactivity.

However, we cannot formally rule out effects mediated by bacterial-derived toxic proteins in our preparation. It is conceivable that suppressive *E. coli*-derived non-protein agents might bind selectively to recombinant *Ov*ALT-2 and *Ov*NLT-1 but not to *Ov*103. These contaminants would not be separated by 3 kDa filtration and would be proteinase-resistant. Thus further structural analysis of the recombinant proteins and especially identification of the putative suppressive peptides generated by proteinase K digestion is needed in order to distinguish bacterial- or protein-specific suppression.

Since we observed a strong suppression of the proliferation of TCR transgenic T cells *in vitro* we asked whether this suppression mimics the inhibitory capacity of nematodes *in vivo*. In a previous study we showed strong suppression of OVA-specific T cell proliferation after adoptive transfer of OT-II T cells in *L* *. sigmodontis* -infected mice [9]. Suppression of OT-II T cell proliferation was less pronounced in mice infected with the intestinal nematode *S* *. ratti* [25]. Different parameters such as site and duration of infection most likely contribute to the magnitude of suppression *in vivo* [35] and might explain the differences in the suppressive capacity of *L* *. sigmodontis* and *S* *. ratti* . As ESP from *O* *. volvulus* strongly suppressed proliferation of OVA-specific T cell *in vitro*, we tested, whether these proteins are sufficient to suppress proliferation of OT-II T cells *in vivo*. After injection of 20 µg of onchocystatin, *Ov*7, we could not significantly dampen the proliferation of OVA-specific T cells *in vivo* after adoptive transfer into C57BL/6 mice. This may reflect the limited effect of short-term administration of suppressive ESP in comparison to the continuous secretion by the living parasite. As our positive control protein did not cause significant suppression of proliferation in this experimental setup, we did not attempt to test the suppressive capacity of the other ESP *in vivo*. To this end, firstly more detailed studies are required concerning the establishment of an ESP application route and regimen sufficient to replace the suppressive capacity of concurrent nematode infection.

Taken together, we identified two *O* *. volvulus* -derived proteins, *Ov*ALT-2 and *Ov*NLT-1, that act as immunomodulators *in vitro* and suppress OVA-specific T cell function. Helminths or their secreted products have been shown to protect against autoimmune diseases and allergies in various mouse models [36]. To identify the active compound is a prerequisite to benefit from the therapeutic potential helminths have due to their immunomodulatory capacity. The role of *Ov*ALT-2 and *Ov*NLT-1 as potential targets in treating autoimmune diseases need to be further addressed in future studies.

## Supporting Information

Table S1 Oligonucleotides used for cloning of the recombinant proteins.
(DOCX)
