# Supplementary material for: Nematode-Derived Proteins Suppress Proliferation and Cytokine Production of Antigen-Specific T Cells via Induction of Cell Death
Source: PLoS One. 2013 Jun 21;8(6):e68380. doi: 10.1371/journal.pone.0068380 (PMC3693813; doi:10.1371/journal.pone.0068380)
Supplement: Table S1 — Oligonucleotides used for cloning of the recombinant proteins. (DOCX) [file pone.0068380.s001.docx]

**Table S1**

**Oligonucleotides used for cloning of the recombinant proteins**

| Primer | Direction | Sequence (5`→3`) | | |
| --- | --- | --- | --- | --- |
| *Ov*NLT-1 | *Nde*I (s) | | | GATACATATGGAAGAAGATTTTGAAGAAGAAGGAGAA |
|  | *Bam*HI *(as)* | | GAGAGGATCCTTAATCATACTTGCAGGTCCAGTTTTC | |
| *Ov*ALT-2 | *Nde*I (s) | | | GATACATATGGAAGATTTTGAAGAAACCGGTGGAGAT |
|  | *Bam*HI (as) | | | TCTCGGATCCTTAATCATATTTGCAGGTCAGTTTTC |
| *Ov*103 | *Nde*I (s) | | | GCCATATGGACTTACTTTCGGAAGCTGG |
|  | *Bam*HI (as) | | | GCGGATCCTTACTCTCGTAAAGTATTG |
| *Ov*7 | *Nde*I (s) | | | GCCATATGAAAAATCCTTCTAAAATGGAA |
|  | *Bam*HI (as) | | | GCGGATCCAACTTCTTTAGTACCAAGAAT |

Restriction sites are underlined, sense (s), antisense (as)
